# Supplementary material for: Occurrence of COVID-19 Symptoms During SARS-CoV-2 Infection Defines Waning of Humoral Immunity
Source: Front Immunol. 2021 Aug 16;12:722027. doi: 10.3389/fimmu.2021.722027 (PMC8416539; doi:10.3389/fimmu.2021.722027)
Supplement: Supplementary file 1 [file DataSheet_1.pdf]

Supplementary Material

1 Supplementary Data

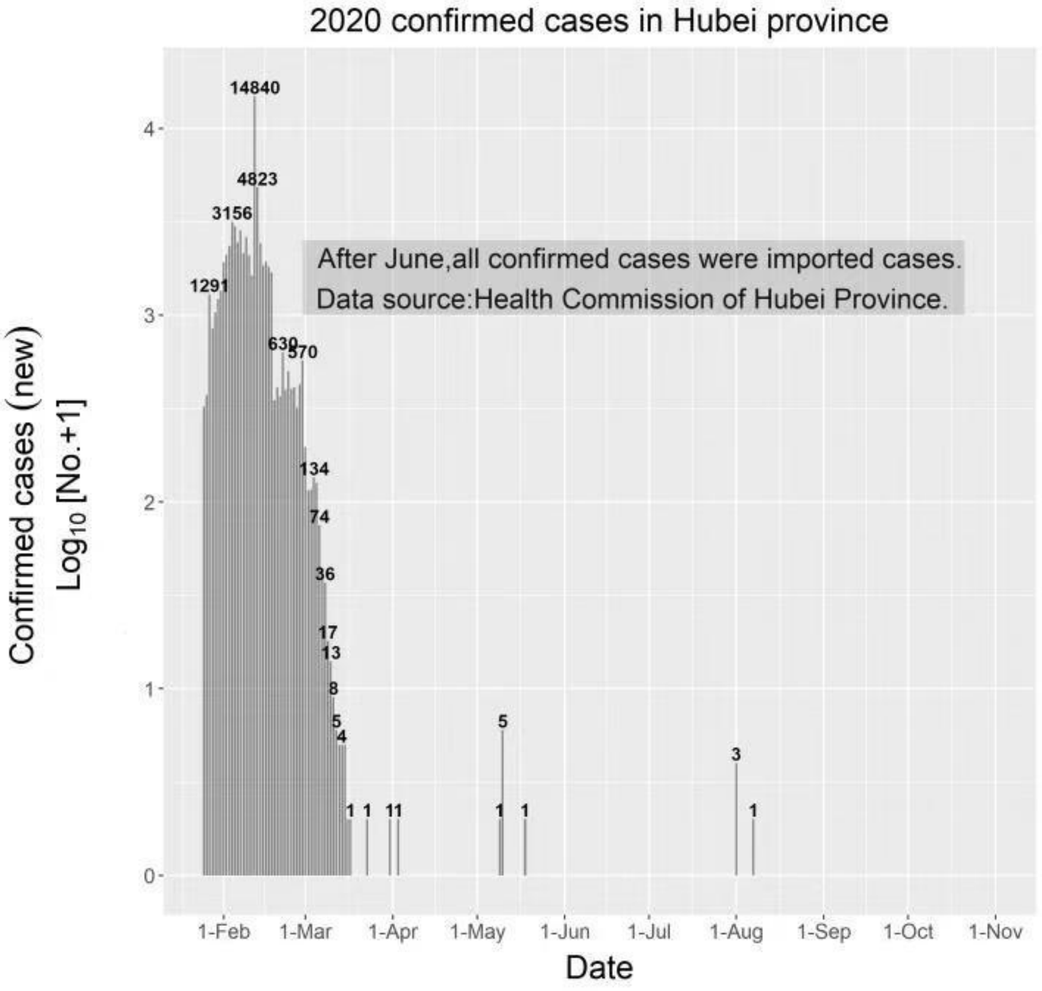

Month	Apr	May	Jun	Jul	Aug	Sep	Oct
Sample [No.]	1196	3149	3410	4552	5456	5656	5758

Supplementary Figure 1. The local spread and the COVID-19 endemic ended before April 1st 2020. The graph depicts newly confirmed SARS-CoV-2 infections in the Hubei province comprising Wuhan according to the public information released by the Health Commission of Hubei Provincial. The table shows the number of samples tested every month.

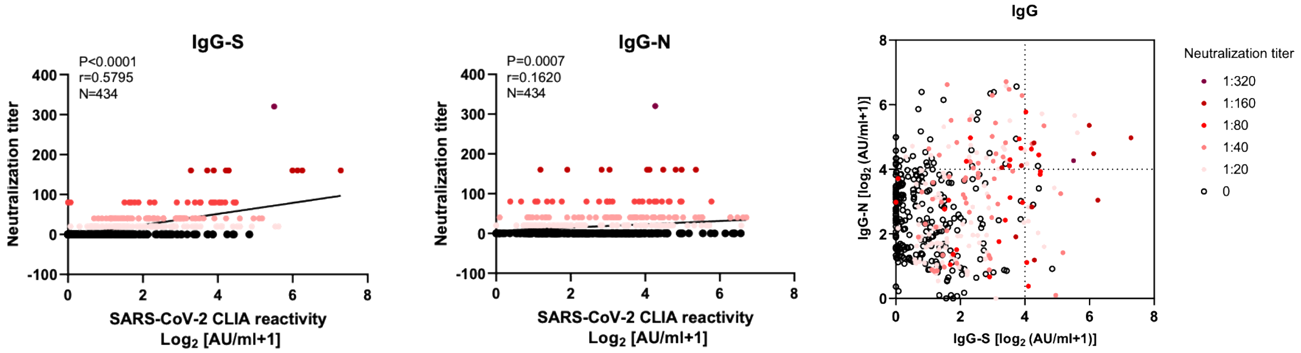

Supplementary Figure 2: Neutralizing antibodies correlate best with IgG recognizing the RBD of the SARS-CoV-2 spike protein. A, Correlation analysis of neutralization titers versus S-specific (left panel) and N-specific (right panel) CLIA-reactive IgG in asymptomatic individuals. A non-parametric Spearman's correlation test was applied for statistical analyses. In the graphs, p, r, and N indicate the p value, the correlation coefficient, and the sample size, respectively. B, Distribution of neutralizing activity as indicated by the colour at different levels of IgG-S (x-axis) and IgG-N (y-axis).

		Total (N=864)	Asymptomatic (N=405)	Symptomatic (N=459)	*P*-value
Gender	Male	387 (44.79%)	195 (48.15%)	192 (41.83%)	0.5052 *
	Female	477 (55.21%)	210 (51.85%)	267 (58.17%)	0.5468 *
Age	Mean (SD)	47 (14)	47 (14)	47 (14)	0.9027 #
	<30	106 (12.27%)	54 (13.33%)	52 (11.33%)	0.6871 *
	30-39	185 (21.41%)	79 (19.51%)	106 (23.09%)	0.5833 *
	40-49	178 (20.60%)	72 (17.78%)	106 (23.09%)	0.4062 *
	50-59	197 (22.80%)	114 (28.15%)	83 (18.08%)	0.1386 *
	>=60	198 (22.92%)	86 (21.23%)	112 (24.40%)	0.6389 *
BMI	Low weight	52 (7.04%)	32 (8.56%)	20 (5.48%)	0.4111 *
	Normal	464 (62.79%)	228 (60.96%)	236 (64.66%)	0.7413 *
	Overweight	205 (27.74%)	107 (28.61%)	98 (26.85%)	0.8132 *
	Obesity	18 (2.44%)	7 (1.87%)	11 (3.01%)	0.6058 *

* Calculated using the χ^2 test.
# Calculated using the unpaired t test.

Supplementary Table 1. Demographic characteristics of asymptomatic individuals and symptomatic COVID-19 patients. All data are presented as median with the interquartile range or as number and percentage. Statistic testing was conducted applying a $\chi 2$ test (marked by *) or a Mann-Whitney U test (marked by #). A p value <0.05 was considered as hallmark of statistical significance.
